# Dosimetric results of whole body irradiation with MOSFET dose tracking to eliminate interfractional variations

**DOI:** 10.1007/s00411-026-01198-8

**Published:** 2026-01-22

**Authors:** Taha Erdoğan, İbrahim Eker, Duriye Öztürk, Özveri Tuğlu, Yeter Düzenli Kar, Nilgün Eroğlu

**Affiliations:** 1 Department of Radiation Oncology Faculty of Medicine, Cancer Institute, Afyonkarahisar Health Sciences University, Zafer Health Complex, Dortyol District, Afyonkarahisar, 03030 Turkey; 2 Department of Paediatric Hematology, Faculty of Medicine, Center, Afyonkarahisar Health Sciences University, Afyonkarahisar, Turkey

**Keywords:** TBI, ALL, HSCT, MOSFET, IVD

## Abstract

Total body irradiation (TBI) is an essential component of conditioning regimens prior to hematopoietic stem cell transplantation (HSCT), particularly in paediatric patients. However, achieving dose homogeneity throughout the treatment course remains a major challenge due to large treatment fields, tissue heterogeneity, and inter-fraction variations. The aim of this study was to investigate the feasibility and impact on dose homogeneity of fraction-based adaptive dose modulation supported by dynamically adjusted rice bag compensators, based on real time metal oxide semiconductor field-effect transistor (MOSFET) feedback, using in vivo dosimetry (IVD) in bilateral TBI. In this context, IVD measurements were performed using MOSFET detectors at the brain, neck, lung, umbilical, and pelvic regions during each treatment fraction in patients undergoing bilateral TBI. Based on these measurements, the thickness of the rice-bag compensators was dynamically adjusted between fractions. Calculated dose values were compared with MOSFET measured doses for each anatomical region. Statistical analysis revealed no significant differences between calculated and MOSFET-measured doses across all anatomical regions (*p* > 0.05), indicating a high level of agreement between planned and delivered doses. The highest dose differences were observed in the lung region (up to 106.9%), whereas the lowest differences were observed in the neck region (up to 100.8%). In conclusion, fraction-based IVD monitoring using MOSFET dosimetry enables adaptive dose modulation in bilateral TBI, reduces inter-fraction dosimetric uncertainties, and provides an effective quality assurance strategy to improve dose homogeneity and treatment safety, particularly in paediatric patients.

## Introduction

In paediatric Acute Lymphoblastic Leukaemia (ALL), conditioning regimens incorporating total body irradiation (TBI) combined with application of etoposide have been shown to be associated with significantly higher overall survival and lower relapse rates compared to chemotherapy-based regimens alone (Peters et al. 2021). Therefore, TBI plus etoposide-based conditioning is recommended for paediatric ALL patients older than four years of age who have an indication for transplantation. TBI is considered an effective treatment option because it maintains efficacy in regions such as the central nervous system, where chemotherapeutic agents may be less effective due to natural barriers, and it is not limited by factors such as drug activation, blood flow, or tumour localization (Gibbons and Khan 2014; Halperin et al. 2019; Mayles et al. 2007). The primary aim of TBI is to eradicate bone marrow and tumour cells and to adequately suppress the immune system prior to bone marrow transplantation (BMT), thereby preparing the patient for transplantation (Gibbons and Khan 2014; Halperin et al. 2019).

Because the planned target volume (PTV) in TBI encompasses the entire body and bone marrow, achieving homogeneous dose distribution presents significant challenges in treatment planning and delivery (Onal et al. 2012). Although various TBI techniques have been developed, there is still no clear consensus regarding the optimal approach. In addition to clinical experience, treatment room geometry and the technical characteristics of the irradiation device play an important role in determining the appropriate TBI technique. Another critical consideration is the need to create a single, large, homogeneous irradiation region to avoid overdose resulting from overlapping adjacent fields. Irregular body contours, tissue heterogeneity, and scatter effects further complicate dose calculation and delivery (Briot et al. 1990). Consequently, the selected TBI technique and dosimetric approach have a direct impact on transplantation outcomes and complication rates (Ramm et al. 2008; Cananoglu et al. 2022).

Currently, TBI is most commonly delivered using conventional linear accelerators (Halperin et al. 2019). However, due to geometric constraints of treatment rooms, there is no standardized approach for source-to-skin distance (SSD) or field size in TBI. In routine radiotherapy, the maximum field size at 100 cm SSD is typically 40 × 40 cm²; however, TBI requires extended SSDs, generally ≥ 300 cm, to adequately cover the large PTV in both paediatric and adult patients (Cananoglu et al. 2022). The prescribed dose is often referenced to the umbilical region due to its relatively stable geometry and low tissue density, and the most commonly used fractionation scheme consists of a total dose of 12 Gy delivered in six fractions (2 Gy per fraction, two fractions per day) (Halperin et al. 2019; Mayles et al. 2007). In conventional practice, dose variations of up to ± 10% from the prescribed dose across different body regions are generally accepted (AAPM  1986).

To improve dose homogeneity and limit radiation toxicity, various optimization techniques have been proposed, including the use of rice bag compensators and organ-shielding blocks for the lungs or kidneys (Bloemen-van Gurp et al. 2007; Schultheiss et al. 2007). The incidence of TBI-related complications is closely associated with dose, with common adverse effects including pulmonary complications, renal toxicity, cataracts, and reduced pituitary function. Among these, interstitial pneumonia remains the most critical and dose-limiting complication of TBI (Sampath et al. 2005; Planskoy et al. 1996).

In this context, in vivo dosimetry (IVD) has emerged as a critical quality assurance tool for measuring, quantifying, controlling, and monitoring dose homogeneity during radiotherapy delivery (Chow and Ruda 2025). Recent in vivo dosimetry studies have also highlighted clinically relevant discrepancies between calculated and measured doses in large-field radiotherapy applications, underscoring the importance of accurate dose verification and real-time monitoring (Demir et al. 2024). Passive dosimeters such as thermoluminescence dosimeters (TLDs) and optically stimulated luminescence dosimeters (OSLDs) are widely used in TBI because of their clinical feasibility and suitability for large-field irradiation (Cananoglu et al. 2022). In addition, OSL-based measurements have been shown to be effective for point-dose verification and quality assurance in radiotherapy applications (Erdogan et al. 2021). However, the lack of real-time dose feedback limits the ability of passive dosimeters to detect and correct dose deviations during treatment.

To overcome this limitation, metal oxide semiconductor field-effect transistor (MOSFET) dosimeters provide a significant advantage by enabling real-time dose measurements, allowing immediate evaluation of fraction-to-fraction dose variations and facilitating adaptive dose management (Bloemen-van Gurp et al. 2007). Owing to their small size and ease of placement in anatomically challenging regions, MOSFET systems are particularly suitable for fraction-based dose monitoring in paediatric TBI. Although MOSFET dosimeters may be more sensitive to cumulative radiation damage than some passive dosimeters, reliable clinical performance can be maintained through regular calibration and quality control procedures (Jong et al. 2018).

The aim of the present study was to investigate the importance of monitoring fraction-to-fraction dose distribution in patients undergoing bilateral TBI by performing MOSFET-based IVD measurements at the brain, neck, lung, abdominal, and pelvic regions during each treatment fraction. In addition, the impact of dynamically adjusting the thickness of rice bag compensators based on real-time MOSFET feedback on the agreement between calculated and measured doses was evaluated, with the goal of optimizing dose homogeneity and improving dose control throughout the treatment course.

To the best of the authors’ knowledge, this is the first clinical study to demonstrate the feasibility of inter-fractional dose correction in TBI based on real-time in vivo dosimetric feedback, rather than post-treatment verification alone. Specifically, unlike previous studies that mainly focused on dose verification after treatment delivery, the present approach actively reduced inter-fraction dose uncertainties during the treatment course.

## Methods

### Selection of patients

Ten paediatric patients (3–16 years old), eight of whom were diagnosed with ALL (80%), one with a diagnosis of Juvenile Myelomonocytic Leukaemia (JMML) (10%) and one with a diagnosis of T-cell lenfoma, who underwent HSCT with the TBI at Afyonkarahisar Health Sciences University (AFSU) Paediatric HSCT Center between 2020 and 2024, were included in the study (Table 1). Measurements were made with MOSFET dosimeters for brain, neck, lung, umbilicus and pelvis regions in each fraction during the entire treatment, to document any inter-fractional variations. The midline dose was calculated from the MOSFET skin doses and then interpreted.

**Table 1:** Sociodemographic characteristics of ten paediatric patients. F – female; M – male; JMML - juvenile myelomonocytic leukaemia; ALL - acute lymphoblastic leukaemia; T-Cell – T-cell Lenfoma

Patient ID	Age (y)	Gender	Diagnosis	Prescribed Dose
1	3	F	JMML	12 Gy
2	4	M	ALL	12 Gy
3	4	M	ALL	12 Gy
4	13	M	ALL	14 Gy
5	16	F	ALL	14 Gy
6	6	F	ALL	12 Gy
7	6	M	ALL	12 Gy
8	13	M	ALL	12 Gy
9	5	M	ALL	12 Gy
10	5	M	T-Cell	12 Gy

### Dosimetry in TBI treatment: calculation formalism and rice bag modulation

For TBI irradiation, it is essential to use a technique that ensures good dose homogeneity and a uniform dose distribution throughout the body. Standard dosimetric data obtained under conditions like a 100 cm SSD and small irradiated areas, common in routine radiotherapy, are not valid for TBI applications, which involve an extended SSD (~ 350 cm) and larger treatment fields (40 × 40 cm²). Therefore, measurements of deep dose distribution and dose profiles should be made specifically under TBI conditions (Mayles et al. 2007). According to Report No. 17 of the American Association of Physicists in Medicine (AAPM), solid water phantoms with dimensions of 30 × 30 × 30 cm³ are considered sufficient for calibration in TBI applications (Kal et al. 2006).

**Fig. 1:**
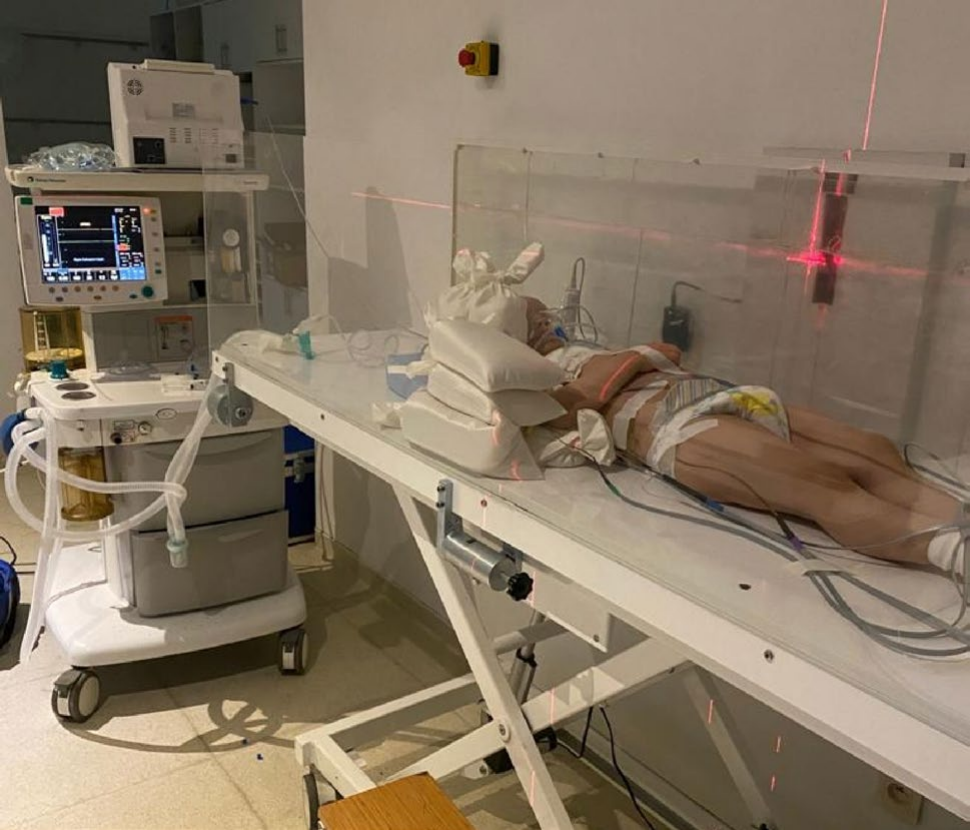
Remote source-to-skin distance (SSD) bilateral total body irradiation (TBI) technique with a single source

The primary step in dose calculation for TBI is determining deep dose distributions along the central axis of the radiation beam. These distributions are characterized by parameters like Deep Dose Percent (DD%), Tissue Air Ratio (TAR), Tissue Phantom Ratio (TPR), and Tissue Maximum Ratio (TMR). Given that TBI is not a standardized treatment, each treatment centre uses its own protocols, which vary based on photon energy, distance, patient positioning, and other technical factors. Once the appropriate TBI technique is selected, horizontal and vertical dose profiles, DD%, and TMR curves should be obtained at the centre of the treatment table. TBI treatments are more complex than routine radiotherapy due to the long patient-source distance, large irradiated area, irregular body contours, varying tissue densities, and patient movement during extended treatments. The chosen technique should ideally protect critical organs, such as the lungs, kidneys, and liver, when necessary. Additionally, the technique should be reproducible and comfortable for fractionated treatments. In the clinic of the authors, a remote SSD bilateral TBI technique using a single source is employed (Fig. 1). The supine position is advantageous for reproducibility and patient comfort; however, achieving dose homogeneity is more challenging than other positions due to the larger lateral width in the supine position (Sampath et al. 2005).

For the present study, patients were positioned supine on a specially designed TBI treatment table, 380 cm from the isocenter (Fig. 1). Treatments were delivered using a 6 MV homogeneous x-ray beam generated by a Varian Trilogy linear accelerator (Palo Alto, CA, USA) at a dose rate of 300 monitor units (MU) per min, with the gantry rotated 90° and the collimator at 45°, producing a 40 × 40 cm² field size and a source-to-axis distance (SAD) of 380 cm. To increase skin dose, a 1-cm scattering plate was placed between the patient and the beam. The total prescribed dose of 12 Gy was delivered using a hyper-fractionated regimen of six fractions, with 2 Gy administered twice daily over three days (Kal et al. 2006). In cases with high disease burden, additional radiotherapy fields may be included in TBI (Peters et al. 2021).

Dose calculations for this study were performed using the classical formalism described by Khan (Eq. 1), incorporating essential parameters such as (TMR), scatter factors, inverse square law correction, off-axis ratio (OAR), and the transmission factor (TF) of the rice bag used for dose modulation.

1 $$D / MU = k \cdot TMR(d, r) \cdot S_{c} (r_{c}) \cdot S_{p} (r_{p}) \cdot (f / f^\prime)^{2} \cdot OAR(d) \cdot TF$$

where *D* is the dose (Gy), *MU* is the monitor unit, *k* is the calibration constant (1 Gy/MU under reference conditions), *TMR(d*,* r)* is the tissue-maximum ratio at depth *d* and equivalent field size *r*, *S*_*c*_ and *S*_*p*_ are the collimator and phantom scatter factors, respectively, *(f/f′)²* is the inverse square law correction, *OAR(d)* is the off-axis ratio at depth *d*, and *TF* is the transmission factor accounting for the rice bag used as a dose modulation material.

**Fig. 2:**
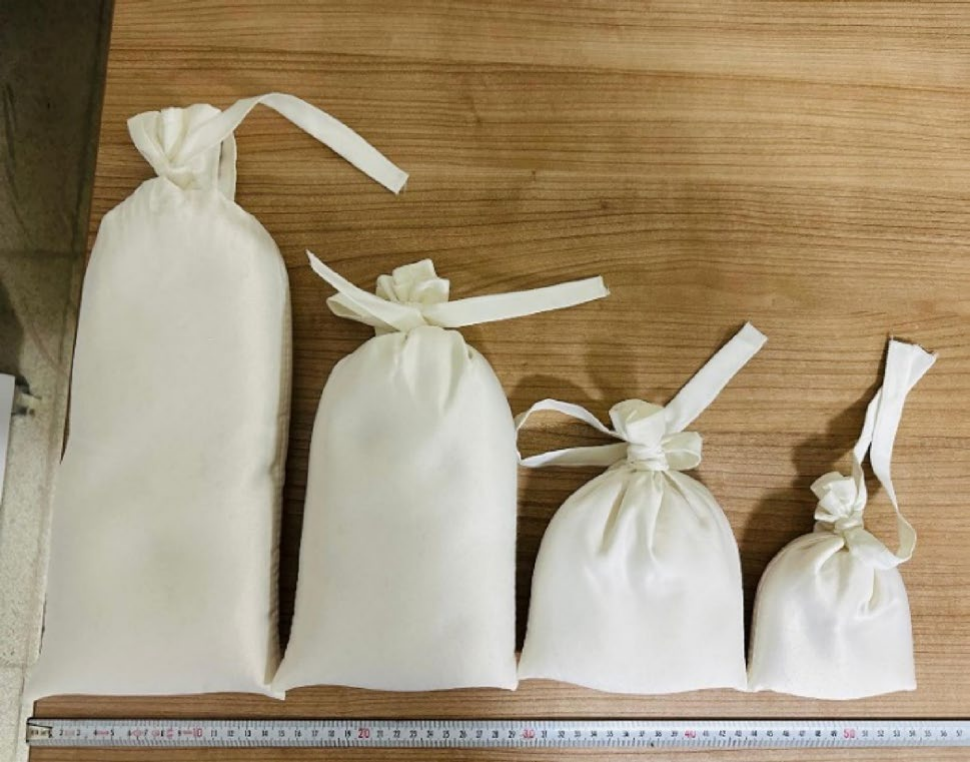
Rice bags with varying thicknesses and densities used for dose modulation

Rice bags with varying thicknesses and densities were used to achieve dose modulation (Fig. 2). TF describes the attenuation effect of the rice bag. It was incorporated into the dose calculation formalism as shown in Eq. (2), where TF is defined as the ratio of the dose measured with the rice bag in place to the dose measured under identical irradiation conditions without the rice bag.

2 $$TF = D_{with\: rice\: bag} / D_{without \:rice\: bag}$$

**Fig. 3:**
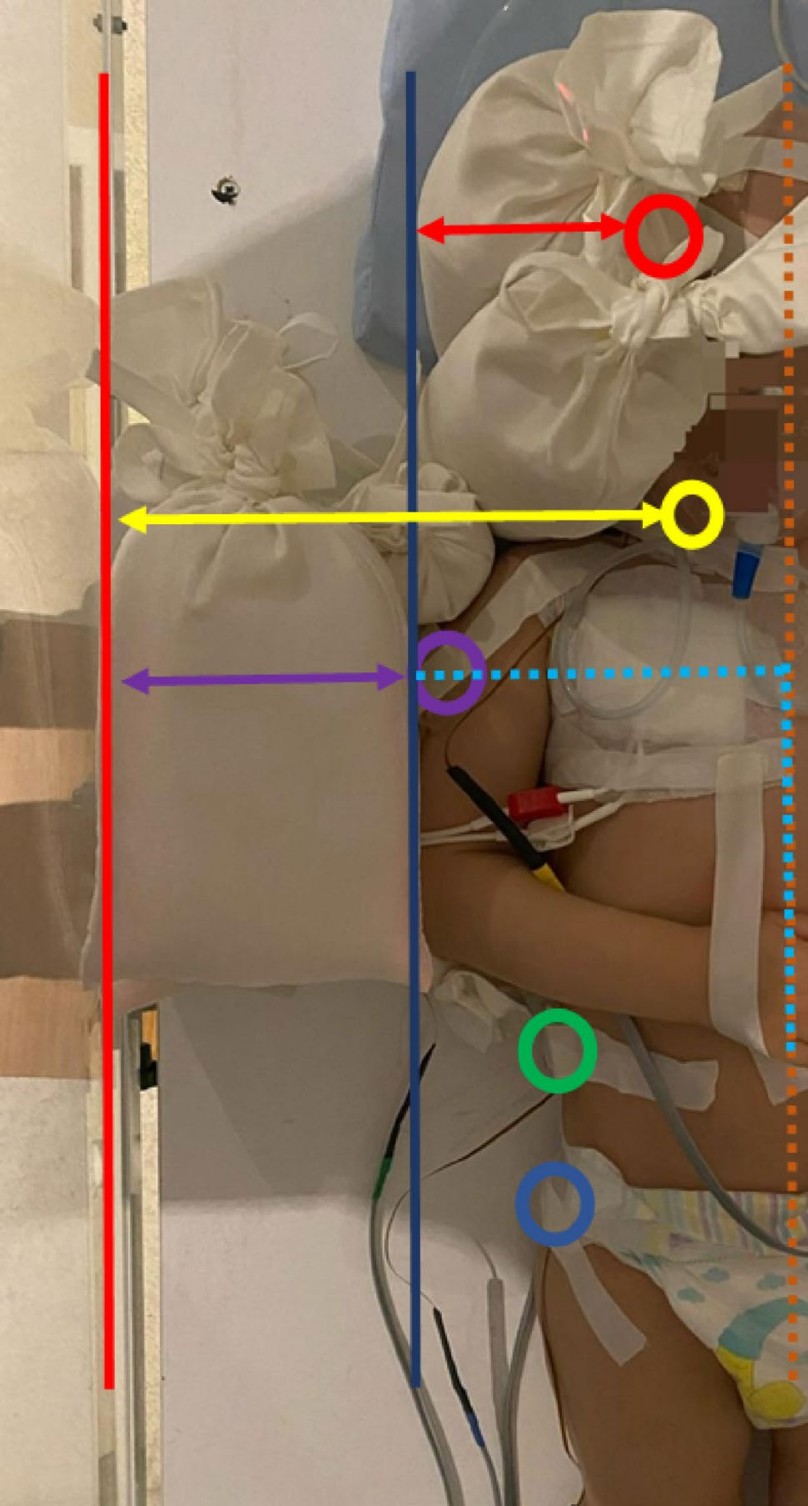
Patient set-up for total body irradiation (TBI), showing in vivo dosimetry (IVD) measurement points and rice-bag thickness references. MOSFET dosimeter markers indicate anatomical measurement sites: head (red), neck (yellow), lung (purple), umbilicus (green), and pelvis (blue). The red vertical line represents the plexi dose-exit plane, while the blue vertical line marks the patient body start reference axis. Transverse arrows denote total rice-bag thickness for the brain (red), neck (yellow), and lungs (purple). The vertical orange dashed line indicates the patient’s midline, and the cyan dashed horizontal line represents the lung tissue maximum ratio (TMR) depth. The dashed horizontal line indicates the off-axis ratio (OAR) depth for the patient’s lung

All TF measurements were performed using a farmer ion chamber under phantom conditions with a fixed source-to-surface distance, field size, and beam energy. The rice bag was positioned on the phantom surface to simulate clinical application, ensuring full field coverage and reproducible placement. These experimentally determined TF values were then used in the dose calculation formalism based on Khan’s approach (Eq. 1) to account for the attenuation and scattering effects introduced by the rice bag. This approach allowed a direct comparison between MOSFET-measured doses and calculated doses under modulated conditions, providing a consistent framework for evaluating the accuracy and reliability of rice bag based dose modulation (Fig. 3).

### In vivo dosimetry (IVD) and treatment monitoring

After determining the parameters to be used in dose calculation, IVD should be performed during the first fraction of each patient, taking into account changes in the patient’s body structure (e.g., weight loss) and position during treatment (Gibbons and Khan 2014). Following the measurement of basic dosimetric data, IVD verification is critical, especially in complex treatments such as TBI, to ensure accurate dose delivery. Previous studies have demonstrated that various dosimetric systems, including TLDs, OSLDs and MOSFET detectors, can be effectively used for patient dose verification in TBI applications (Briere et al. 2008). Continuous monitoring of the delivered dose is vital in such complex treatments. MOSFET dosimeters were selected for in vivo dose measurements because their real-time readout capability allows immediate evaluation of dose deviations and supports adaptive dose management during the TBI treatment course. The prescribed dose should be controlled by a real-time dose tracking system to prevent deviations. In TBI, in-vivo dosimetry usually measures the reference point dose at the mid-pelvis or mid-abdomen and the midline doses at different points in the craniocaudal direction. The doses of organs at risk, such as the lungs and liver, are also monitored (Benyunes et al. 1995). The doses delivered to the patient during treatment are compared to the calculated dose values. IVD monitoring allows for correction of dosimetric errors between fractions and prevents the propagation of these errors to subsequent fractions. This helps reduce potential side effects and ensures dose homogeneity within ± 10% of the target dose (Kal et al. 2006; AAPM Report No. 17).

### Calibration and usage protocol of MOSFET dosimeters

**Fig. 4:**
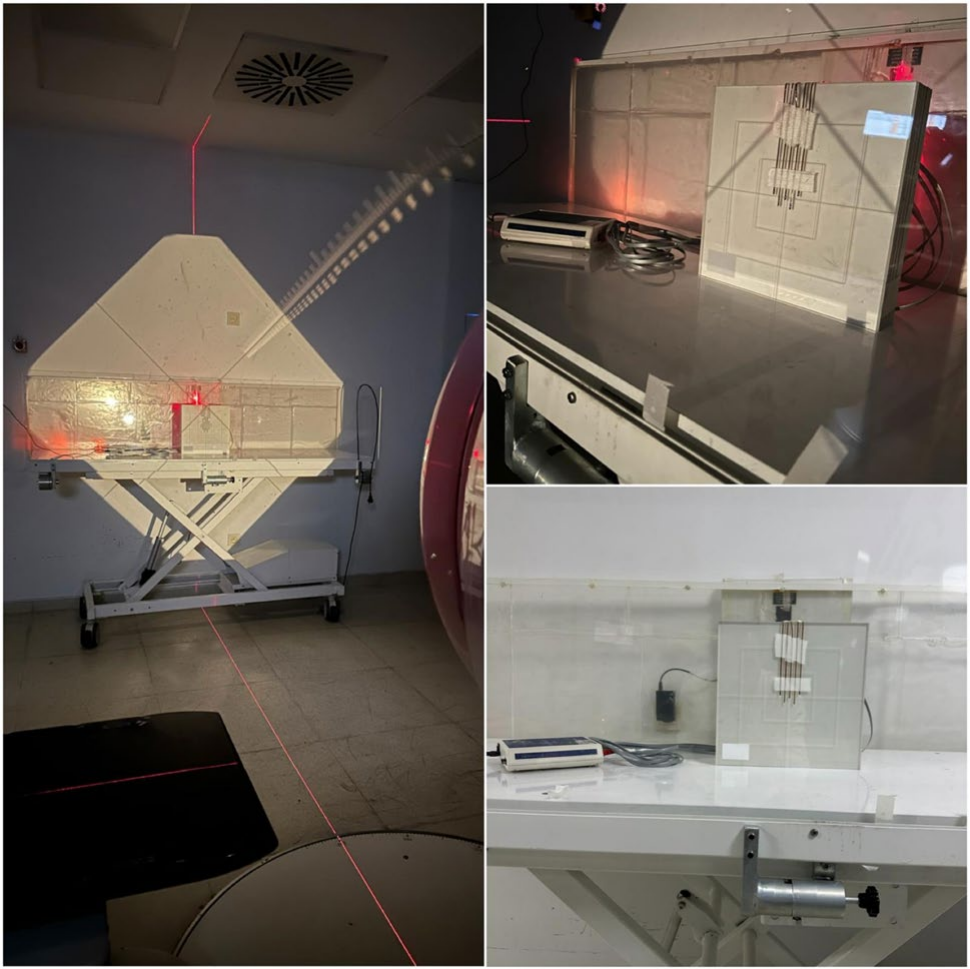
MOSFET dosimeters positioned on a solid water phantom during the calibration procedure

According to the AAPM Task Group-29, verification of the dose delivered in TBI requires absolute dose calibration to be performed at the treatment distance using water or water-equivalent phantoms under large-field TBI irradiation conditions. In the present study, solid water phantoms were used for calibration, and MOSFET dosimeters were positioned at appropriate locations on the phantom (Fig. 4). The MOSFET detectors were calibrated on a Varian Trilogy linear accelerator using a 6 MV photon beam, delivering a dose of 2 Gy under lateral beam geometry. While performing the calibration, the SAD distance of the TBI protocols and a 1 cm thick scattering plate were used. Dosimetric uncertainties that could arise from calibration performed under patient-simulated setup conditions were minimized by using this approach. In treatment setups, the MOSFET dosimeters were wrapped in a 1-cm thick bolus due to the build-up effect and attached to the patient’s skin. When calculating the midline dose, the bolus thickness wrapped in the MOSFET dosimeters was also taken into account. Signals were checked with a wireless mobile MOSFET reader (Model TN-RD-16, Thomson-Nielson, Denmark) and remote dose verification software running on a laptop. Necessary compensator revisions were made between the set-ups according to the midline dose values obtained, and the most homogeneous dose distribution was tried to be achieved in the subsequent set-ups.

### Statistical analyses

The comparison between measured and calculated dose values was performed using the Mann–Whitney U test for cases with rice bag modulation, and the Wilcoxon signed-rank test for cases without modulation. Statistical significance was considered at *p* < 0.05. All statistical analyses were conducted using SPSS version 20.0 (IBM Corp., Armonk, NY, USA).

## Results

**Fig. 5:**
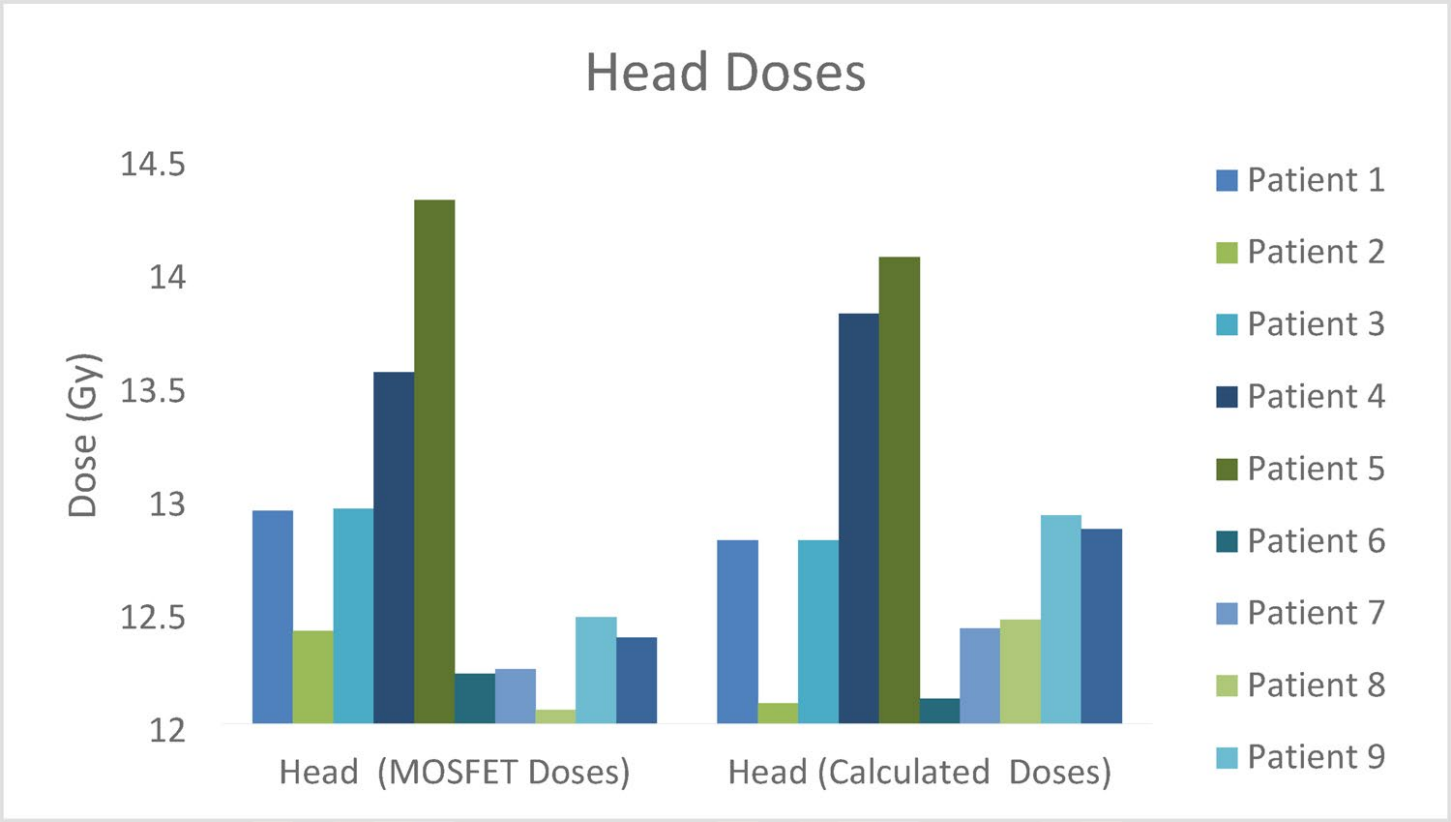
Calculated and MOSFET head midline doses of the TBI treatment applied

**Fig. 6:**
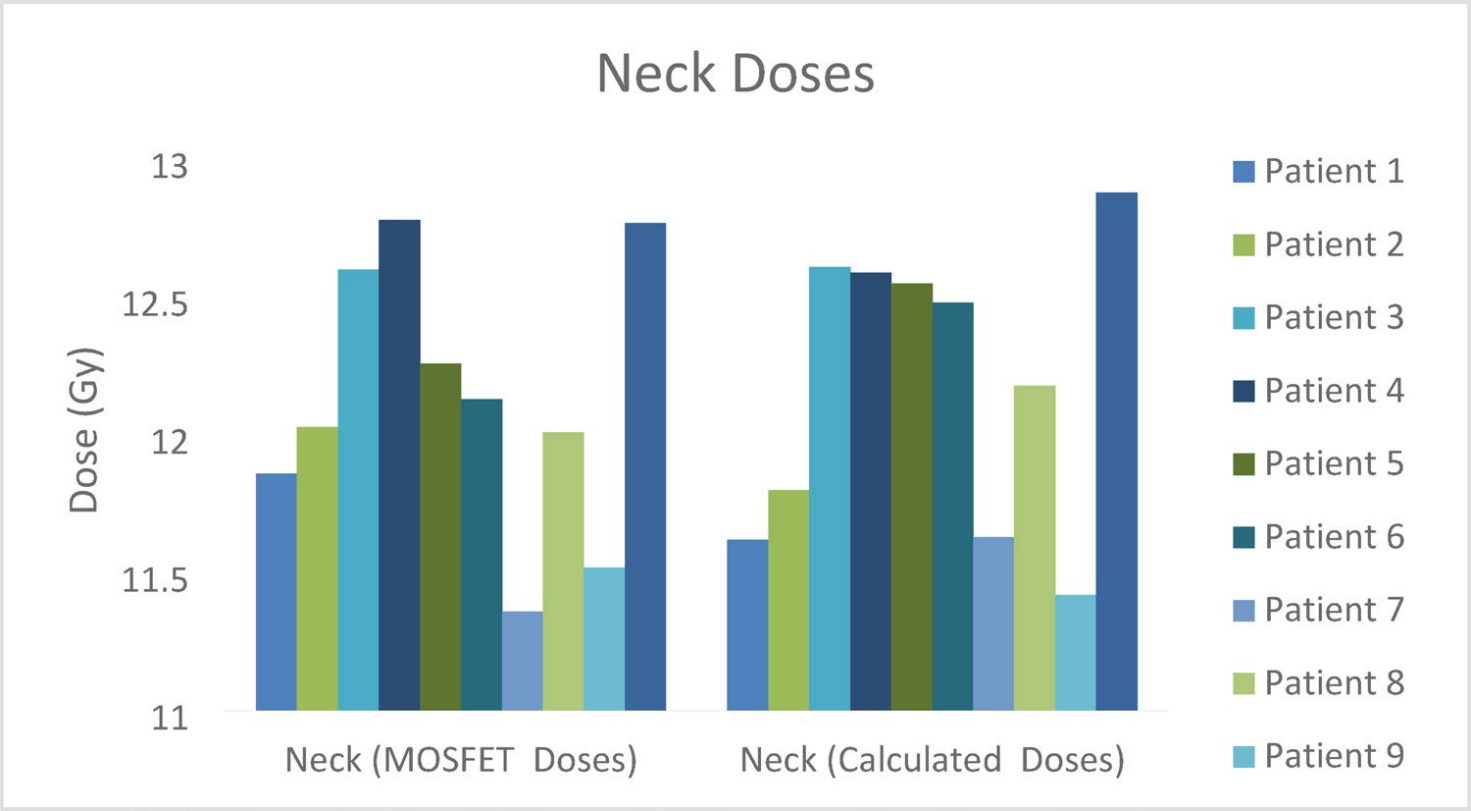
Calculated and MOSFET neck midline doses of the TBI treatment applied

**Fig. 7:**
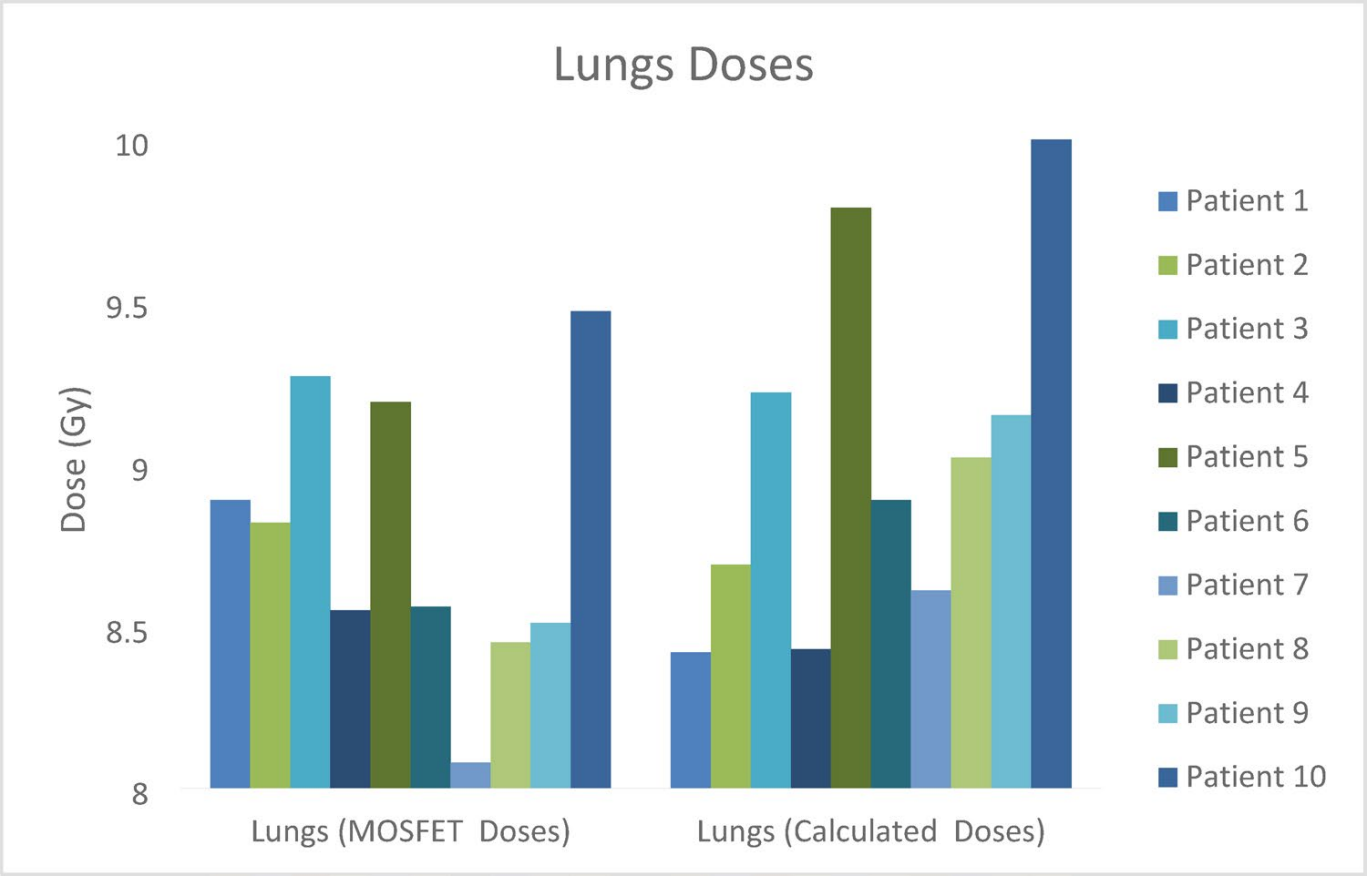
Calculated and MOSFET lungs midline doses of the TBI treatment applied

**Fig. 8:**
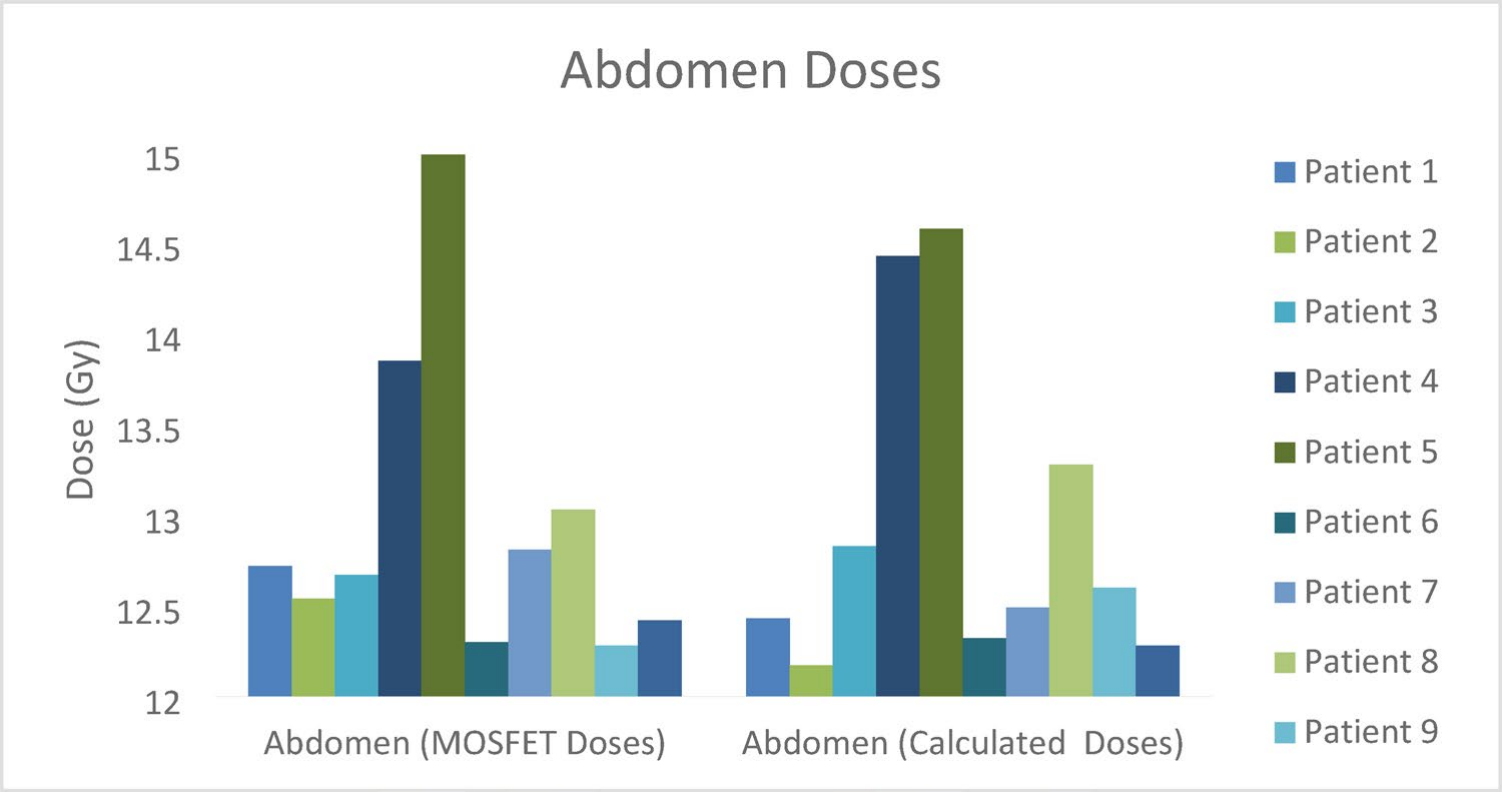
Calculated and MOSFET abdomen midline doses of the TBI treatment applied

**Fig. 9:**
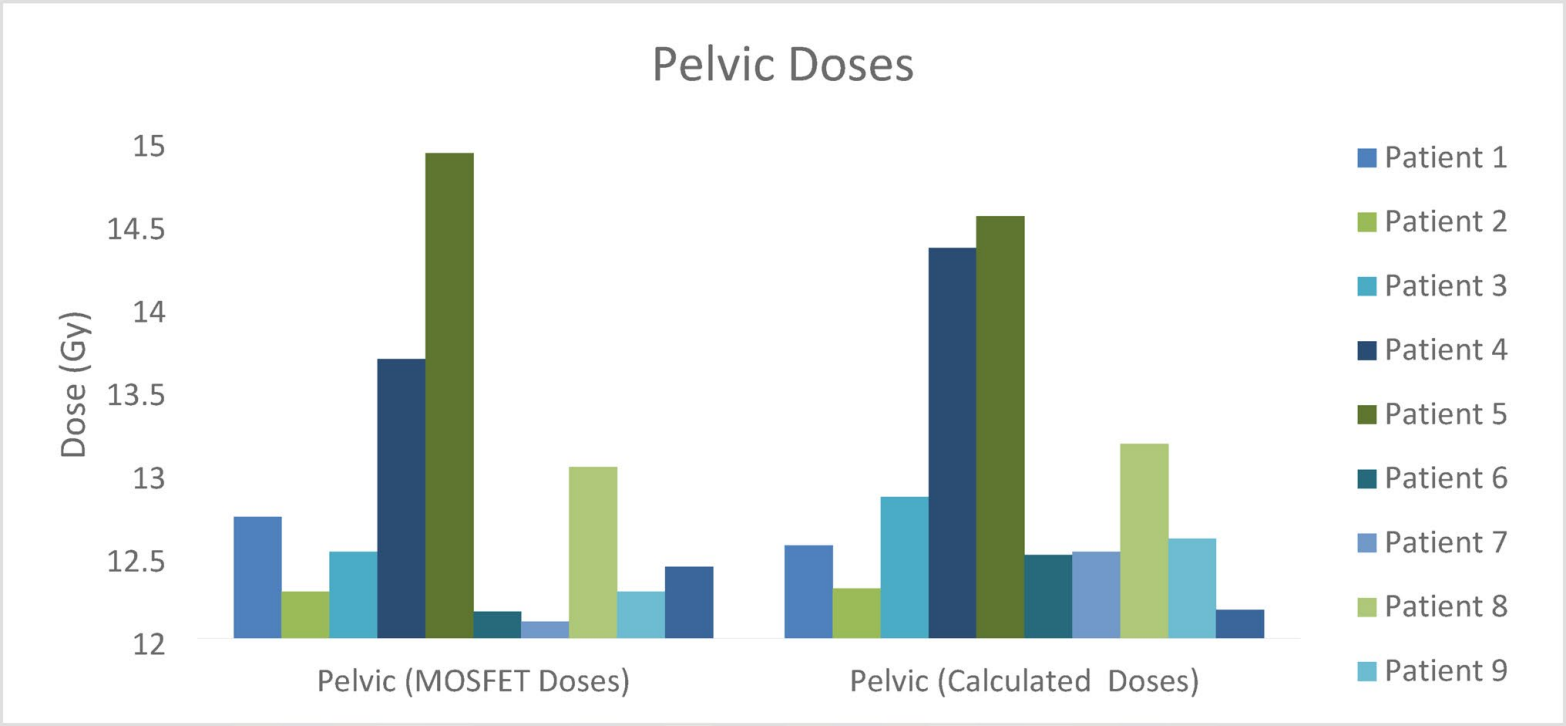
Calculated and MOSFET pelvic midline doses of the TBI treatment applied

**Fig. 10:**
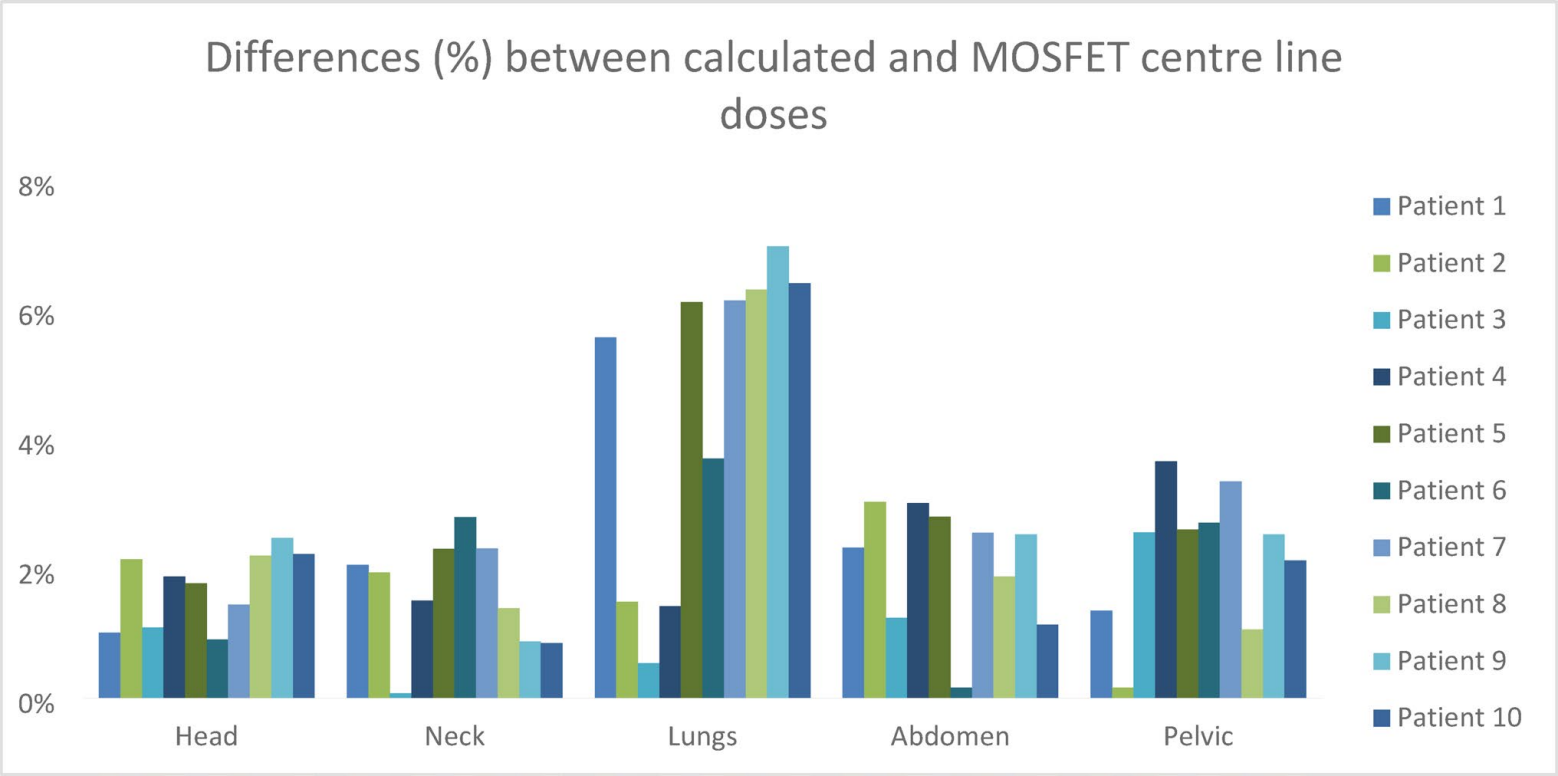
Differences (%) calculated and MOSFET cumulative midline doses of the TBI treatment applied

The regional comparison of calculated and measured MOSFET midline doses of TBI treatment performed is shown in Figs. 5, 6, 7, 8 and 9 and the differences (%) between cumulative midline doses are shown in Fig. 10.

**Fig. 11:**
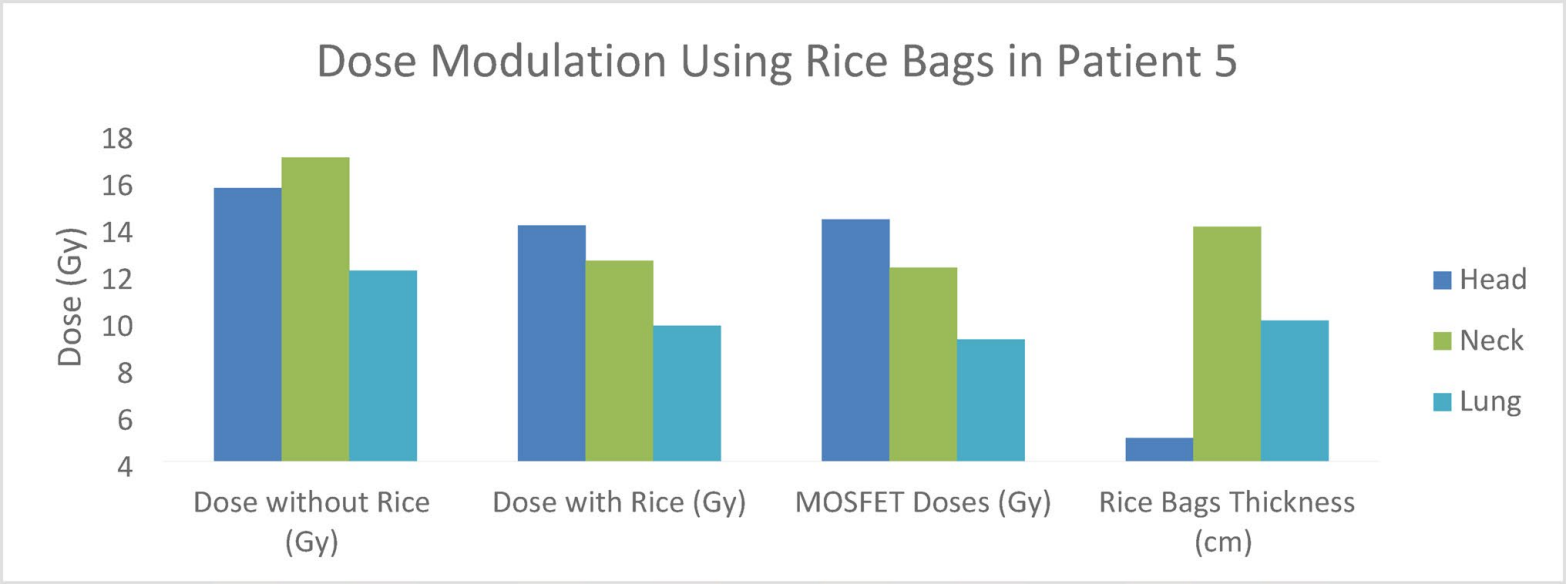
Comparison of calculated and MOSFET-measured midline doses across different anatomical regions during total body irradiation with rice bag–based dose modulation for Patient 5, with a total prescribed dose of 14 Gy

## Discussion

In this study, the reliability of patient-based dose modulation performed using rice bags was evaluated by comparing MOSFET IVD measurements with dose values calculated by the authors using a formula defined in the literature. This dose modulation approach, applied for the first time in the literature for TBI patients, was implemented interfractionally using rice bags. By reducing the average effect of dose fluctuations between fractions, this approach contributed to maintaining agreement between MOSFET measurements and calculated dose values. Previous studies in radiotherapy have reported that interfraction interventions improve dose stability and enhance measurement calculation agreement, particularly in anatomical regions involving near-surface measurement points and the buildup region (Chow and Leung 2008; Jong et al. 2018).

Unlike previous TBI studies that primarily focused on post-fraction dose verification, the present study introduced a fraction-based adaptive dose modulation strategy in which rice-bag compensators are dynamically adjusted based on real-time MOSFET IVD. This approach enables active reduction of inter-fraction dose uncertainties and represents a novel application of MOSFET-based IVD in bilateral TBI. This approach allows TBI treatments to be delivered safely, rapidly, and effectively in paediatric patients. Moreover, its ease of implementation with existing equipment makes it a practical and efficient method for bilateral TBI treatments.

Preparative regimens including TBI are particularly preferred in cases of ALL, as TBI can reach sanctuary sites such as the gonads and the central nervous system that may not be adequately treated by chemotherapy alone. When selecting a TBI technique, factors such as photon beam energy, dose homogeneity, maximum achievable treatment field size, treatment distance, dose rate, patient dimensions, setup comfort, and the need to spare certain normal tissues to reduce side effects must be carefully considered (Gibbons and Khan 2014). As demonstrated by the clinical outcomes of the present study, the use of the bilateral TBI technique provides acceptable dose homogeneity and clinically reasonable dose levels. In addition, when compared with advanced techniques such as Field-In-Field (FIF), Volumetric Arc Modulated Radiotherapy (VMAT), Tomotheraphy and Image-Guided Radiotherapy (IGRT), the bilateral TBI technique requires shorter simulation, contouring, treatment planning, and treatment times, and offers easier setup. The longer setup and treatment durations associated with these advanced techniques may lead to dose distribution deterioration due to patient motion, field overlap issues, and potentially increased risks of toxicity or secondary malignancies. Therefore, particularly in paediatric patients treated under anesthesia, time-efficient techniques appear to be more appropriate. Furthermore, acquiring computed tomography (CT) images and delivering treatment using the same respiratory support devices increase the workload in TBI techniques, which are already inherently complex.

In the present study, the most commonly used treatment regimen reported in the literature, i.e., 12 Gy delivered in six fractions was applied (Gibbons and Khan 2014), and in two patients requiring additional dose, one fraction of 2 Gy was prescribed (Kal et al. 2006). For all patients and all measurement points, the differences between calculated and measured doses remained within the ± 10% homogeneity criterion, consistent with clinically accepted tolerance limits for TBI (Kal et al. 2006; AAPM Report No. 17).

In the head region, differences between calculated and measured doses were observed to be approximately within the range of 1–2% (Figs. 5 and 10). Similarly, dose differences in the neck region mostly remained within 1–3% (Figs. 6 and 10). These relatively low differences may be explained by the more homogeneous tissue density in the head and neck regions and the ability to position MOSFET detectors more reproducibly and stably in these areas as compared to other body regions. In addition, the reduced interpatient variability in the thickness of rice bags used for dose modulation in these regions contributed to improved agreement between calculated and measured doses. Previous studies have reported high agreement between MOSFET-based IVD measurements and TPS calculations in anatomically homogeneous regions (Onal et al. 2012).

In TBI, the lungs represent the most critical dose-limiting organ due to their radiosensitivity. Furthermore, given the high risk of fungal infections in the lungs of these patients, lung dose is of particular importance in TBI applications. The most common pulmonary toxicity associated with TBI is radiation pneumonitis. According to AAPM Report No. 17, lung dose in TBI treatment plans should not exceed 80–85% of the prescribed dose (Kal et al. 2006). While maintaining lung dose constraints, it should also be considered that excessively low lung doses may compromise treatment efficacy and reduce the level of immunosuppression required for successful bone marrow transplantation. In conventional fractionation schemes delivered twice daily (1.5–2 Gy per fraction), no significant increase in pneumonitis risk has been reported for total doses up to 15 Gy (Halperin et al. 2019). In the present study, dose differences between calculated and measured values in the lung region were higher than those observed in other anatomical regions, ranging approximately from 3.5% to 7% (Figs. 7 and 10). The low density of lung tissue, the presence of air tissue interfaces, and the use of large radiation fields in lateral TBI geometry are major factors limiting dose calculation accuracy. Similarly, the AAPM Report No. 17 emphasizes that measured doses in heterogeneous tissues may exhibit greater deviations from TPS calculations.

In the abdominal region, dose differences between calculated and measured values were mostly within the range of 2–3% (Figs. 8 and 10). Although the abdomen contains relatively homogeneous soft tissue, small variations in patient positioning, respiratory motion, and millimetric deviations in MOSFET detector placement may have contributed to these differences. Dose discrepancies of this magnitude in the abdominal region during TBI are considered clinically acceptable in the literature (Kal et al. 2006).

In the pelvic region, dose differences between calculated and measured values were found to range approximately between 2% and 4% (Figs. 9 and 10). Increased tissue thickness, pronounced bone soft tissue heterogeneity, and minor patient-specific variations in the thickness of rice bags used for dose modulation may have contributed to these deviations. It is well known that dose calculations in thick and heterogeneous anatomical regions are more sensitive to geometric uncertainties than thinner and more homogenous anatomical regions (Khan 2014).

Statistical analyses further support the effect of rice bag based dose modulation on dose stabilization. According to the Mann–Whitney U test, no statistically significant differences were observed between MOSFET measurements and calculated doses in the head (*p* = 0.762), neck (*p* = 0.880), lung (*p* = 0.450), umbilical (*p* = 0.734), and pelvic (*p* = 0.212) regions when rice bag modulation was applied. This indicates good agreement between calculated and measured doses under interfraction dose modulation. In contrast, Wilcoxon signed-rank test results obtained without rice bag modulation revealed statistically significant differences in the head (*p* = 0.001), umbilical (*p* = 0.000), and pelvic (*p* = 0.000) regions, while no significant differences were observed in the lung (*p* = 0.313) and neck (*p* = 0.287) regions. The lack of statistical significance in the lung region may be attributed to higher measurement and calculation uncertainties caused by tissue heterogeneity, which may mask systematic differences. Overall, these findings suggest that dose consistency decreases in the absence of dose modulation, particularly in anatomically variable and geometrically sensitive regions, whereas rice bag modulation significantly contributes to interfraction dose stabilization. The data presented in Fig. 11 reveal a clear inverse relationship between rice bag thickness and midline dose. The use of rice bags was observed to produce more substantial dose reductions in anatomically thinner regions, while dose variations remained relatively constrained in regions with greater effective thickness. These findings suggest that rice bags constitute an effective dose modulation approach for compensating anatomical thickness variations and improving dose uniformity in total body irradiation. Although this trend is illustrated using a representative patient, similar patterns of dose modulation were consistently observed across all treatment fractions analyzed in the study.

Previous studies have reported potential discrepancies between dose calculations and IVD measurements in anatomical regions involving near-surface measurement points and the buildup region, and have shown that interfraction interventions can improve dose stability (Chow and Leung 2008; Jong et al. 2018). Similarly, studies investigating the use of modulation or bolus materials have demonstrated improved agreement between TPS calculations and measured doses, particularly in near-surface and heterogeneous regions, along with reduced measurement uncertainty (Gül et al. 2024). These findings support the contribution of rice bag based dose modulation to interfraction dose stabilization in near-surface dose regions during bilateral TBI applications.

Overall, discrepancies between calculated and measured doses were higher in regions containing heterogeneous tissues, particularly the lungs, and lower in homogeneous and geometrically stable regions such as the head and neck (Fig. 10). These findings demonstrate that MOSFET-based IVD is an important safety tool for interfraction dose monitoring in bilateral TBI applications and that rice bag based dose modulation can be implemented within clinically acceptable limits. This approach provides a significant advantage, especially in paediatric patients, by enabling early detection of interfraction dose deviations and timely adjustment of dose modulation when necessary.

The present study has several limitations. Although MOSFET dosimeters offer advantages for paediatric patients and anatomically challenging regions due to their small size and real-time measurement capability, their sensitivity to cumulative radiation exposure, as well as energy and angular dependence, may contribute to measurement uncertainty (AAPM Report No. 17; Rosenfeld 2005; Butson et al. 2000). Percentage differences between measured and calculated doses may be associated with small variations in detector placement, uncertainties in transmission factors, and incomplete electronic equilibrium in near-surface anatomical regions (AAPM 1986 ; Khan 2014). In addition, dose calculations were based on a single formula-based approach, which required certain geometric and material properties to be evaluated under idealized assumptions. These assumptions may limit dose calculation accuracy, particularly in heterogeneous tissue regions and large-field geometries. Nevertheless, the absence of statistically significant differences between measured and calculated doses indicates that the applied calculation approach is clinically valid and applicable under real treatment conditions (Kal et al. 2006; Khan 2014).

## Conclusions

This study demonstrated, for the first time, the clinical feasibility of inter fractional dose modulation using rice bags in bilateral TBI, evaluated through MOSFET-based IVD. The close agreement between measured and calculated doses, with deviations remaining within established clinical tolerance limits, indicates that rice bag modulation effectively stabilizes dose delivery between fractions. The absence of statistically significant differences when modulation was applied further supports its role in improving inter fractional dose consistency.

This approach is particularly advantageous in paediatric patients, where reduced treatment time, setup simplicity, and motion sensitivity are critical considerations. Without the need for complex planning techniques or additional equipment, rice bag based dose modulation provides a practical, cost effective, and reproducible solution for achieving clinically acceptable dose homogeneity in bilateral TBI. These findings support its safe integration into routine clinical practice, especially in resource limited settings.

## Data Availability

No datasets were generated or analysed during the current study.
